# Technology-mediated Chinese learning beyond the classroom: the role of L2 resilience and L2 engagement

**DOI:** 10.3389/fpsyg.2026.1920084

**Published:** 2026-09-23

**Authors:** Huijuan Hou, Chenrui Miao, Jingyao Shi

**Affiliations:** 1 School of Law, Chongqing University, Chongqing, China; 2 School of Education, Curtin University, Perth, WA, Australia; 3 University International College, Macau University of Science and Technology, Taipa, Macao SAR, China

**Keywords:** Chinese as a second language, L2 engagement, L2 resilience, language learning beyond the classroom, self-regulated learning, technology literacy

## Abstract

**Introduction:**

Out-of-class autonomous language learning is an important dimension of second language (L2) development, particularly in higher education contexts where learners are expected to regulate their own learning beyond formal instruction. In technology-mediated learning environments, this autonomy increasingly depends on learners' technology literacy (TL), understood not merely as the ability to operate digital tools but as a self-regulated capacity to select resources, evaluate information, solve learning problems, and sustain participation in digital learning contexts. In Chinese as a Second Language (CSL) learning, however, relatively little is known about CSL learners' TL in autonomous out-of-class learning or about the learner-side factors that may support this capacity.

**Methods:**

This mixed-methods study examined the current profile of TL among 327 international CSL learners in mainland China and investigated whether L2 resilience and L2 engagement predicted TL. Quantitative data were collected through questionnaire surveys and analyzed using descriptive statistics, correlation analysis, multiple regression, and robustness checks. Qualitative data were collected through semi-structured interviews with six participants and analyzed thematically.

**Results:**

Learners reported a moderate level of TL. Both L2 resilience and L2 engagement significantly and positively predicted TL, with L2 engagement showing the stronger predictive effect. Interview findings further indicated that learners used technology to support examination preparation, maintain Chinese exposure, access authentic digital communities, and connect Chinese learning with academic, professional, and everyday life needs.

**Discussion:**

These findings suggest that TL in out-of-class CSL learning is closely connected with learners' persistence, engagement, and self-regulated use of digital resources. The study offers implications for helping CSL learners select, combine, and sustain technology-mediated Chinese learning practices beyond the classroom.

## Introduction

Optimal language learning depends not only on in-class engagement but also on learning beyond the classroom (LBC), which has become an increasingly important condition for sustained language development and academic achievement (Benson, 2011; Reinders and Benson, 2017). In higher education contexts, LBC is particularly significant because learners are expected to assume greater responsibility for regulating their own learning, selecting appropriate resources, and maintaining language exposure beyond formal instruction. However, out-of-class learning does not automatically lead to effective language development. Without clear instructional supervision, learners may experience reduced motivation, fragmented attention, uncertainty about learning strategies, or difficulty sustaining effort over time (Dong, 2025).

In the past decade, technological development has substantially reshaped the nature of out-of-class foreign language learning. Digital tools, online platforms, social media, learning applications, and AI-assisted resources have enabled learners to access authentic input, communicate with wider language communities, and design more personalized learning trajectories (Butler, 2022; Lai et al., 2024). In this sense, technology-mediated LBC is not merely an extension of classroom learning, but a self-regulated learning environment in which learners must decide what tools to use, how to use them, and how to evaluate whether these tools contribute meaningfully to their language development. Thus, the value of technology in L2 learning depends not only on the availability of digital resources, but also on learners' capacity to use such resources strategically and persistently.

Technology Literacy (TL) is therefore a crucial construct in technology-mediated language learning. In the present study, TL is understood not simply as the operational ability to use digital devices or online platforms, but as a technology-mediated self-regulated learning capacity. This capacity involves locating resources, learning how to use unfamiliar tools, evaluating the quality of online information, managing distractions, interacting through digital media, and applying technological resources to solve language learning problems (Lopez and Arabadzhy, 2026). In autonomous out-of-class contexts, TL is especially demanding because learners must simultaneously manage technological challenges and disciplinary language-learning challenges. For example, learners may need to master a new application, judge whether online Chinese materials are reliable, or use digital tools to overcome difficulties in vocabulary, listening, writing, or sociocultural communication (Vuorikari et al., 2022).

From this perspective, L2 resilience and L2 engagement provide two theoretically relevant predictors of TL. In technology-mediated out-of-class learning, resilience is important because learners may encounter technical barriers, confusing resources, limited feedback, or slow language progress (Wang et al., 2025). Learners with stronger resilience are more likely to continue experimenting with tools, adjust their strategies, and use technology to cope with problems rather than abandon the learning process when difficulties arise (Chen X. et al., 2025).

L2 engagement, by contrast, reflects learners' emotional, behavioral, and cognitive participation in language learning (Wang et al., 2026). Because TL cannot be developed through occasional or superficial tool use, sustained engagement is necessary for learners to transform technological access into actual learning capacity. Learners who are more engaged are more likely to invest time in repeated practice, explore digital resources actively, participate in online communication, and reflect on how technology supports their Chinese learning (Wang and Wang, 2024). In this sense, engagement represents the behavioral and cognitive enactment of self-regulated learning, while resilience supports persistence when this process becomes difficult (Wang and Wang, 2024).

In the field of Chinese as a Second Language (CSL), TL has become increasingly important because Chinese learners often rely on technological tools to connect classroom instruction with out-of-class practice. Prior research has shown that Chinese learners benefit from digital resources, learning platforms, social media, and technology-supported learning systems that facilitate Chinese learning across formal and informal contexts (Gu et al., 2019). Nevertheless, existing CSL research has paid limited attention to learners' TL in autonomous out-of-class learning. More importantly, little is known about how psychological and behavioral factors, particularly L2 resilience and L2 engagement, are associated with learners' ability to develop and use TL in self-directed Chinese learning.

To address these gaps, this study investigates technology-mediated Chinese learning beyond the classroom by examining the relationship among TL, L2 resilience, and L2 engagement. Specifically, the study explores the current profile of CSL learners' TL, tests whether L2 resilience and L2 engagement predict TL, and further examines how learners use technological tools to navigate difficulties and sustain engagement in autonomous Chinese learning. By conceptualizing TL as a technology-mediated self-regulated learning capacity, this study aims to clarify why resilience and engagement matter for out-of-class CSL learning and to provide pedagogical implications for supporting learners' strategic, persistent, and meaningful use of technology.

## Language beyond classroom (LBC)

Language Beyond the Classroom (LBC) is broadly understood as informal or non-formal second language (L2) learning that takes place outside formal classroom instruction, including learners' engagement with language-related resources, communities, and practices beyond scheduled lessons (Tragant et al., 2021). Within this conceptualization, LBC is regarded as a metacognitive learning approach that emphasizes the importance of learning awareness, the transferability and diversity of English learning environments, and the value of personalized learning experiences (Reinders et al., 2022).

LBC also provides learners with opportunities to participate in wider language-use communities beyond formal instruction (Wagner, 2004). Through self-directed selection of learning materials and communicative partners, learners construct positive interpersonal relationships within shared language spaces, express perspectives on socially relevant topics, and encourage interactive orders within a contacting community—thereby acclimating to personalized public identity (Richards, 2015). Such autonomous learner identity reflects a shift from teacher-directed learning toward learner-directed participation, resource selection, and meaning-making. Learners transition in this process from being passive recipients of linguistic knowledge to becoming active integrators. This shift in roles directly aligns with Norton (2001) “imagined identity theory”. By establishing autonomous learning spaces in their private, out-of-class environments, learners exercise agency and gain greater control over their learning process, thereby cultivating a dynamic, future-oriented self-image as language users. Darvin and Norton (2021), alongside Al-Hoorie et al. (2021), consistently maintain that the imagined identity is founded on the learner's belief that investing substantial effort will yield positive academic returns. By affirming the value of out-of-class learning and emotionally satisfying the need for meaningful practice, this identity organically connects the learner's past, present, and future language study, thereby reinforcing their sense of subjective responsibility for their language learning.

Historically, research concerning LBC has predominantly focused on single qualitative studies. The primary contributions of this qualitative work lie in consistently confirming learners' recognition of the importance and irreplaceability of out-of-class language learning, alongside comprehensively documenting individual, specific learning experiences outside the classroom. The lack of corresponding quantitative research, however, makes it challenging to rigorously examine how out-of-class language study precisely impacts the cognitive and affective components of language acquisition.

Another significant limitation is the paucity of research investigating LBC across diverse settings. Currently, in the CSL field, only a handful of pioneering qualitative studies have explored the ways and experiences of learners autonomously studying Chinese in varying environments (Ramsay, 2003; Linnell, 2001). Thus, it is equally imperative to adopt an integrative perspective to examine the specific LBC learning experiences of learners from different language backgrounds and how these experiences impact their language learning outcomes. In this sense, LBC is not merely a physical extension of classroom learning, but a self-regulated learning space in which learners must actively select resources, monitor progress, and sustain participation without immediate teacher supervision.

## Technology literacy (TL)

Long before the recent emergence of large language models (LLMs) such as Chat GPT and Deep Seek, technology literacy (TL) had already attracted attention in technology-mediated language learning (Hofmeyr, 2023). The main impetus for this focus stemmed from instructors' acute awareness of the limited instructional time within the classroom. In particular, large class sizes often further restrict opportunities for frequent teacher-learner interaction or for guiding learners into somewhat group interactions (Lees and Lorentzen, 2023). Concurrently, due to the limited classroom time, instructors rarely dedicate it to large-scale drills and practice, preferring instead to channel these activities into autonomous learning via post-class assignments. Instructors also view this as an opportunity to help learners engage with more authentic language environments, as opposed to the semi-simulated communicative scenarios often led by the teacher in the classroom (Mahmud, 2018). In the present study, TL is understood not merely as the operational ability to use digital tools, but as a technology-mediated self-regulated learning capacity through which learners locate resources, evaluate information, select appropriate tools, manage online learning processes, and apply digital support to solve language-learning problems. This understanding is consistent with recent digital competence and digital literacy frameworks, which emphasize information evaluation, problem solving, communication, content creation, and responsible participation in digital environments (Ng et al., 2021; Vuorikari et al., 2022). By enriching their exposure to the foreign language through diverse sources, learners enhance their differentiated communicative competence, strengthen their multidimensional cognitive processing of specific topics, and thereby achieve improved learning efficiency and reinforce the extensibility and effectiveness of classroom instruction (Rajabalee et al., 2020).

Research on digital literacy, digital competence, and technology-integrated language learning has expanded rapidly in recent years. Recent studies have increasingly highlighted the role of digital literacy and technology integration in supporting personalized language learning, learner autonomy, and sustained participation in technology-mediated learning environments (Ma, 2025; Zou et al., 2025).

For students initially entering higher education, prior studies have primarily centered on how TL influences learners‘academic emotions, Willingness to Communicate (WTC), and academic achievement (Botes et al., 2025), as well as the establishment of technology-assisted English learning communities through affective and cognitive instructional strategies (Rasikawati et al., 2024). Given that TL is still a relatively edging research domain within ESL and requires a guiding macro-framework, studies targeting postgraduate TL education have mainly observed this group's perspectives on technology integration in learning task design (Mali, 2024), how they utilize technology to complete academic interactions during task performance (Anas et al., 2024), how the application of technological tools enhances authentic English academic performance (Yu and Xie, 2025), and whether learners experience fluctuations in self-efficacy and motivation during academic tasks (Song, 2024). Furthermore, numerous studies are now beginning to incorporate the Technology Acceptance Model (TAM) to investigate the TL acceptance rate among postgraduates (Ma, 2025) and empirically observe how foreign language learners leverage their TL to achieve desired pedagogical outcomes in specific language skills (Zou et al., 2025; Wang and Reynolds, 2024). With such robust empirical surveying, many researchers have put forth recommendations for pre-service English teachers on how to cultivate learners' TL competencies (Rafidiyah et al., 2024) and have offered advice to in-service teachers on managing challenges arising from TL deficiencies and technological drawbacks (ElJishi, 2024; Nakagawa, 2024).

In the CSL field, technology integration has also become increasingly visible (Lyu and Qi, 2020), although research has focused more on instructional design and tool affordances than on learners' own TL in autonomous learning contexts. Currently, CSL has established four promising paradigms for TL cultivation: personalized and adaptive learning systems, including chat bots, Chinese instructional apps, and Chinese learning consortia; social interaction platforms, such as We Chat and I Talki; immersive Chinese learning environments built upon Chinese films and television series; and a range of formal instructional resources, including MOOCs, sanctioned by the Chinese national government (Ge et al., 2012).

However, the intensive and rapid development of TL resources within CSL simultaneously raises critical questions: the actual accessibility of technological tools for learners, whether learners‘TL has truly improved, and, crucially, if their current TL is sufficient to effectively manage such an abundance of technological resources. Within the context of technology-enhanced learning, several issues must be urgently addressed by the CSL educational community, including L2 Chinese learners' ability to access and filter information, strategically apply tools, create and interact with content, and engage in technological metacognitive management (Qing and Jing, 2024). Otherwise, TL in CSL risks devolving from a learner-centered pedagogical design into an uncontrolled teacher-initiated celebration. Key questions remain to further investigate: Can learners accurately match technology to their learning needs instead of mindlessly following the technology tools? Can they develop the integrated capacity to use a combination of technologies when a single tool proves insufficient? And can they shift from passively consuming technological resources to actively mastering them? These questions are prominent to determining whether learners can genuinely leverage technology to enhance their Chinese learning outcomes outside the classroom (Eubanks et al., 2017). However, relatively little mixed-methods research has examined whether CSL learners possess the TL needed to use these resources effectively in autonomous out-of-class Chinese learning, or how affective and behavioral factors support this capacity.

## L2 academic resilience (AR)

Academic resilience (AR) is commonly understood as learners' capacity to sustain effort, adapt to difficulty, and maintain academic functioning in the face of setbacks (Chen X. et al., 2025). In technology-mediated LBC contexts, this capacity is especially relevant because learners often need to solve technical problems, evaluate unfamiliar resources, and continue learning without immediate teacher support.

From an essential perspective, L2 resilience primarily encompasses how learners mobilize metacognitive strategies in the face of stressful and challenging foreign language environments to mitigate resistance to foreign language learning and enhance their sense of achievement derived from it. This suggests that the core characteristics of L2 resilience lie in its adaptability and long-term dynamic nature. Richardson (2002) resilience model helps explain how learners recognize difficulties, develop coping strategies, and implement adaptive responses over time.

The first stage is the Identification Stage. During this stage, learners observe their emotional and academic states when confronting adversity and explore viable means to counter the negative impact of setbacks, thereby increasing their sense of control and self-efficacy toward the L2. The second stage is the Skill Development Stage. L2 learners synthesize the emotional and academic fluctuations experienced in the first stage and begin formulating plans to establish an effective set of strategies for coping with L2 frustrations or stress. The third stage is the Implementation Stage. In this final stage, L2 learners actively tackle various potential difficulties, and through this ongoing process, continuously cultivate their resilience and adjust their academic strategies.

In the context of autonomous out-of-class Chinese learning, L2 resilience becomes even more critical (Liu et al., 2022). Resilience signifies an individual's ability to persistently strive toward goals; to appropriately regulate academic emotions when encountering setbacks or feeling academically fatigued; to specifically seek external assistance when genuinely facing difficulties; and to be sustained by a strong belief in self-efficacy, maintaining the conviction that all effort invested will ultimately yield returns. Currently, as an emerging construct within the field of L2 emotion research, the main focus of L2 resilience studies centers on its relationship with self-efficacy, motivation, and engagement (Wang et al., 2025), while also focusing on the influence of L2 enjoyment on resilience (Aghayani et al., 2024). In an out-of-class L2 Chinese learning environment where direct instructor support is absent, L2 resilience may serve as an important protective factor for maintaining motivation, self-efficacy, and engagement in autonomous Chinese learning. Despite this, research is still lacking on how L2 resilience interacts with TL, and explorations in this specific area within the CSL domain are particularly insufficient.

## L2 engagement

Skinner and Pitzer (2012) conceptualized L2 engagement in educational contexts as “constructive, enthusiastic, willing, emotionally positive and cognitively focused participation with learning activities in the classroom” (p. 22). This view suggests that engagement is not limited to observable participation, but also includes learners' emotional investment and cognitive focus during learning activities (Li et al., 2025). This enjoyment, in turn, signifies more than a fleeting sense of pleasure; it reflects the learner's belief that they are engaged in a meaningful, self-directed activity, one that contributes to a shared, sustained, and personally referenced network of academic effort—one defined by internal benchmarks rather than social comparison (Fine and Corte, 2017).

While L2 motivation typically represents a learner's individual willingness to invest in a specific learning task or activity, L2 engagement, building upon this foundation, truly represents the learner's authentic performance during the learning process (Afzali et al., 2021). L2 engagement refers to learners' sustained behavioral participation, emotional involvement, and cognitive effort during language-learning activities. Crucially, language proficiency improvement necessitates extensive language interaction and practical tasks, achieved through the learner's proactive, meaningful linguistic output and interaction with others (Azila-Gbettor et al., 2022). A unique function of L2 engagement in foreign language learning is its direct and decisive influence on the effectiveness of L2 instruction and learning. Without sufficient engagement, the effectiveness of instructional materials, technological tools, and pedagogical support is likely to be limited. Undeniably, L2 engagement can be built, and its cultivation primarily follows the three dimensions proposed by Fredricks et al. (2004): cognitive engagement, emotional engagement, and behavioral engagement. These three dimensions are particularly relevant to TL because technology-mediated learning requires repeated tool use, active problem solving, emotional persistence, and sustained cognitive investment.

Consequently, recent research concerning L2 engagement has consistently proceeded from these three dimensions. In the area of Cognitive Engagement, researchers have primarily investigated its relationship with L2 engagement through the lenses of critical thinking (Ma and Xu, 2025), perceived teacher support (Li, 2024), and teacher credibility (Alipour and Modarresi, 2024). For Emotional Engagement, studies have explored its correlation with L2 engagement through constructs such as mindfulness, L2 control, L2 self-esteem (Chen L. et al., 2025), L2 enjoyment, L2 boredom, L2 anxiety (Ma and Xu, 2025), and L2 resilience (Wang and Wang, 2024). In the domain of Behavioral Engagement, researchers have mainly examined how factors like teacher assessment (Bui et al., 2025) and feedback (Fu and Huang, 2025) influence L2 engagement.

However, relatively little research has examined how L2 engagement functions in autonomous out-of-class learning, particularly in relation to learners' TL in CSL contexts. Furthermore, there is a lack of investigation into the relationship between emotional engagement and behavioral engagement. Finally, current studies are still heavily concentrated on the ESL context, warranting an examination of L2 engagement across diverse language learning environments.

## Current study

Based on this review, the present study conceptualizes TL as a technology-mediated self-regulated learning capacity and examines whether L2 resilience and L2 engagement function as affective and behavioral predictors of this capacity in autonomous CSL learning.. Therefore, this paper proposes three research questions (RQs):

1. What is the current profile of Technology Literacy (TL) among Chinese as a Second Language (CSL) learners in autonomous out-of-class Chinese learning?
2. To what extent do L2 resilience and L2 engagement predict CSL learners' TL?
3. How do CSL learners use technological tools to navigate difficulties and sustain engagement in self-directed Chinese learning beyond the classroom?

## Method

### Participants

A total of 327 international students enrolled at three universities in mainland China participated in the questionnaire survey. These universities were selected because they all offered Chinese language-related courses to international students and provided access to learners with different academic backgrounds and Chinese learning experiences. Among the participants, 268 were undergraduates and 59 were postgraduates. All participants were non-native speakers of Chinese and had experience of using digital tools or online resources for Chinese learning beyond the classroom. Questionnaire data were collected through Wenjuanxing (https://www.wjx.cn/), a widely used online survey platform in mainland China. After screening for completeness and validity, 327 valid responses were retained for quantitative analysis.

In the second phase, six participants were invited to take part in semi-structured interviews. The interviewees were selected from the questionnaire participants through purposive sampling to capture variation in gender, academic backgrounds, Chinese Proficiency, and technology-mediated learning experience. The purpose of the interviews was not to achieve statistical representativeness, but to provide explanatory qualitative evidence for interpreting the questionnaire findings. Table 1 presents the demographic information of the six interview participants.

**Table 1:** Demographic information of the qualitative participants.

Number coding	Academc backgrounds	Gender	Chinese proficiency	Technology-mediated Chinese learning experience
Student A (SA)	BA in applied linguistics	Female	HSK-3	Yes
Student B (SB)	BA in engineering	Male	HSK-5	Yes
Student C (SC)	BA in economics	Male	HSK-4	Yes
Student D (SD)	PhD in sociology	Female	HSK-6	Yes
Student E (SE)	Business students	Male	HSK-4	Yes
Student F (SF)	MA in applied linguistics	Male	HSK-6	Yes

### Instruments

Three adapted questionnaire scales were used to measure Technology Literacy (TL), L2 resilience, and L2 engagement. The TL scale was adapted from Huang et al. (2021), which was originally developed to measure students' technology literacy. Although the original scale targeted junior high school students, its core dimensions, including information access, tool use, digital communication, resource evaluation, and technology-supported problem solving, were considered relevant to the present context of autonomous out-of-class CSL learning. To improve contextual appropriateness, items were revised to refer specifically to Chinese learning beyond the classroom and to learners' use of digital tools, online resources, and technology-mediated learning environments. A pilot study was conducted with 14 CSL learners who did not participate in the main study. The pilot aimed to examine item clarity, contextual relevance, completion time, and preliminary reliability. Based on participants' feedback, minor wording adjustments were made to improve clarity and to ensure that the items referred consistently to out-of-class Chinese learning. The pilot reliability of the TL scale was high, with a Cronbach's alpha coefficient of 0.91. These 14 learners will not participate in the subsequent main study.

L2 resilience was measured using an adapted version of the 10-item Connor-Davidson Resilience Scale (CD-RISC-10; Campbell-Sills and Stein, 2007). Items were contextualized to reflect learners' persistence, coping, and emotional regulation in Chinese learning. Following the administration of the questionnaire to the 14 learners, the resulting Cronbach's alpha coefficient was 0.86.

L2 engagement was measured using an adapted version of Lin and Huang (2017) curriculum engagement scale. Because the original scale focused on formal course engagement, the items were revised to capture learners' behavioral, emotional, and cognitive engagement in autonomous Chinese learning beyond the classroom. The Cronbach's alpha coefficient after the 14 participants completed the questionnaire was 0.89.

For the qualitative component, researchers employed semi-structured interviews, utilizing a set of pre-designed core questions while also incorporating supplementary exploratory questions based on learners‘individual experiences and personalized, emergent narratives. This approach allowed the researchers to maintain consistency across interviews while also exploring participants' individual experiences in greater depth. All interviews were conducted online via Tencent Meeting (VooV Meeting) and were approximately 20 min in duration. Participants were permitted to use either Chinese or English for the interview. A total of four participants chose to be interviewed in Chinese, and two chose English. The English interviews were transcribed verbatim and, where necessary, translated into Chinese for coding and comparison by an English instructor from a local university with over 20 years of teaching experience.

### Data analysis

This study followed an explanatory sequential mixed-methods design, in which questionnaire data were first analyzed quantitatively and interview data were then used to explain and contextualize the quantitative findings. For quantitative data analysis, SPSS 31.0 was used. Descriptive statistics, including means, medians, standard deviations, and score distributions, were first calculated to describe the current profile of CSL learners' TL. Because the TL scale included multiple dimensions, dimension-level scores were also compared to examine whether apparent differences among dimensions were statistically meaningful. Pearson correlation analysis was then conducted to examine the bivariate relationships among TL, L2 resilience, and L2 engagement. Multiple regression analysis was subsequently performed to test the predictive effects of L2 resilience and L2 engagement on TL. Before interpreting the regression model, assumptions were examined, including linearity, multicollinearity, independence of residuals, homoscedasticity, and the distribution of residuals. Model fit, regression coefficients, confidence intervals, and diagnostic indices were then reported.

For qualitative data analysis, thematic analysis was conducted with the assistance of NVivo 15. The analysis followed two stages. In the first stage, open coding was used to identify meaningful units and emergent codes from the interview transcripts. In the second stage, related codes were compared, grouped, and refined into broader themes that reflected participants' technology-mediated Chinese learning experiences beyond the classroom. The coding was completed by the first author, and the second author conducted secondary coding on 80% of the initial codes. Disagreements were discussed and resolved through negotiation, resulting in a final coding consistency of 0.95.

The quantitative and qualitative findings were integrated at the interpretation stage. Specifically, the interview themes were used to explain, elaborate, and contextualize the quantitative results, particularly the observed relationships among TL, L2 resilience, and L2 engagement. This integration was consistent with the explanatory sequential logic of the mixed-methods design, in which qualitative evidence was used to provide a deeper understanding of the questionnaire findings.

## Results

### Preliminary analyses: reliability, validity, and regression diagnostics

Before addressing the research questions, preliminary analyses were conducted to examine scale reliability, distributional properties, the factorial structure of the adapted measures, and the assumptions underlying the regression model. Because the TL measure was adapted from an Internet literacy scale originally designed for junior high school students, pilot reliability was not treated as sufficient evidence of its appropriateness for the present CSL learner sample. Therefore, the main-sample analysis reported internal consistency, corrected item-total correlations, KMO values, Bartlett's tests of sphericity, and exploratory factor analysis (EFA) results.

Table 2 presents the descriptive statistics, distributional properties, and reliability coefficients for the three main variables. The three focal scales demonstrated high internal consistency. Cronbach's alpha was 0.971 for TL, 0.948 for L2 resilience, and 0.974 for L2 engagement. Corrected item-total correlations were strong across the three scales, indicating that the retained items contributed consistently to their respective total scores.

**Table 2:** Descriptive statistics, distributional properties, and reliability of the main variables.

Variable	*N*	*M*	SD	Skewness	Excess kurtosis	Cronbach's α
Technology literacy	327	3.26	1.05	−0.59	−1.13	0.971
L2 resilience	327	3.23	1.03	−0.45	−1.17	0.948
L2 engagement	327	3.33	1.02	−0.64	−1.03	0.974

Kurtosis values are reported as excess kurtosis.

Table 3 presents the preliminary factor-analytic results for the adapted scales. The KMO values were 0.980 for TL, 0.964 for L2 resilience, and.982 for L2 engagement, and Bartlett's tests of sphericity were significant for all three scales (*p* < 0.001), indicating that the data were suitable for factor analysis. The EFA results suggested a dominant general factor within each scale. The first factor explained 67.28% of the variance in TL, 68.09% in L2 resilience, and 66.59% in L2 engagement.

**Table 3:** Preliminary factor-analytic results for the adapted scales.

Scale	KMO	Bartlett's test of sphericity	First factor variance explained
Technology literacy	0.980	*p* < 0.001	67.28%
L2 resilience	0.964	*p* < 0.001	68.09%
L2 engagement	0.982	*p* < 0.001	66.59%

EFA, exploratory factor analysis. The first factor variance explained refers to the percentage of total variance accounted for by the dominant general factor in each scale.

Accordingly, the main quantitative analyses used total scores for TL, L2 resilience, and L2 engagement. The five TL dimensions were retained only for descriptive interpretation, and no strong claims were made about them as distinct latent dimensions without further confirmatory evidence.

### Descriptive results for CSL learners' technology literacy

To address the first research question, descriptive statistics were used to examine the current profile of TL among CSL learners. TL was scored after reverse-coding Q6–Q8, which were negatively worded items in the supplementary questionnaire. The mean TL score was 3.26 on a five-point scale (SD = 1.05), indicating a moderate level of self-reported TL. This result suggests that CSL learners in the present sample were not generally characterized by low TL; rather, their reported ability to use technological resources for Chinese learning was slightly above the scale midpoint.

Table 4 presents the descriptive statistics for the five theory-based TL dimensions. The dimension-level means were closely clustered, ranging from 3.25 to 3.29. Specifically, the mean scores were 3.27 for Knowledge and Skills for Internet, 3.25 for Internet Self-management, 3.25 for Cognition of Internet, 3.29 for Internet Interactions, and 3.27 for Autonomous Learning on Internet. Given the small differences among these means and the EFA results reported above, these five dimensions are interpreted only as theory-based descriptive categories rather than as empirically distinct latent dimensions in the present dataset.

**Table 4:** Descriptive statistics of the theory-based TL dimensions.

Dimension	*N*	*M*	SD	Skewness	Excess kurtosis	Cronbach's α
Knowledge and Skills for Internet	327	3.27	1.11	−0.56	−0.98	0.879
Internet Self-management	327	3.25	1.13	−0.55	−0.95	0.847
Cognition of Internet	327	3.25	1.07	−0.58	−0.97	0.901
Internet Interactions	327	3.29	1.11	−0.51	−0.94	0.843
Autonomous Learning on Internet	327	3.27	1.12	−0.47	−1.00	0.847

TL, Technology Literacy. Kurtosis values are reported as excess kurtosis. The five dimensions are retained for descriptive interpretation only.

### Correlation analysis

Pearson correlation analysis was conducted to examine the bivariate associations among TL, L2 resilience, and L2 engagement. As shown in Table 5, TL was significantly and positively correlated with both L2 resilience (*r* = 0.486, *p* < 0.001) and L2 engagement (*r* = 0.543, *p* < 0.001). L2 resilience and L2 engagement were also positively correlated (*r* = 0.510, *p* < 0.001). These results suggest that learners who reported higher resilience and engagement also tended to report higher TL in autonomous out-of-class Chinese learning.

**Table 5:** Correlations among TL, L2 resilience, and L2 engagement.

Variable	1	2	3
1. Technology literacy	—		
2. L2 resilience	0.486^***^	—	
3. L2 engagement	0.543^***^	0.510^***^	—

TL; Technology Literacy. ^***^p < 0.001.

### Regression diagnostics

Regression diagnostics were assessed at the model level, with a focus on residual-based assumptions rather than the marginal normality of each observed variable. As shown in Table 6, multicollinearity was not a concern, as the VIF values for both L2 resilience and L2 engagement were 1.352. The Durbin-Watson statistic was 1.968, suggesting no obvious autocorrelation in the residuals. The Breusch-Pagan test was not significant (LM = 0.286, *p* = 0.867; *F* = 0.142, *p* = 0.868), indicating that the homoscedasticity assumption was acceptable.

**Table 6:** Regression diagnostic results.

Diagnostic	Statistic	*p*	Interpretation
VIF: L2 resilience	1.352	—	No serious multicollinearity
VIF: L2 engagement	1.352	—	No serious multicollinearity
Durbin-Watson	1.968	—	No obvious residual autocorrelation
Breusch-Pagan LM	0.286	0.867	Homoscedasticity acceptable
Breusch-Pagan F	0.142	0.868	Homoscedasticity acceptable
Residual Shapiro-Wilk	0.954	< 0.001	Some departure from residual normality
Jarque-Bera	20.482	< 0.001	Some departure from residual normality

VIF, variance inflation factor. HC3 robust standard errors and bootstrap confidence intervals with 5,000 resamples were used as robustness checks because residual normality tests were significant.

Residual normality tests were significant (Shapiro-Wilk W = 0.954, *p* < 0.001; Jarque-Bera = 20.482, *p* < 0.001), suggesting some departure from normality. Given the sample size and the residual normality results, HC3 robust standard errors and bootstrap confidence intervals based on 5,000 resamples were used as robustness checks. These robustness checks supported the same substantive interpretation as the main OLS model.

### Results of Multiple Linear Regression (MLR)

A multiple linear regression was conducted to examine whether L2 resilience and L2 engagement predicted CSL learners' TL. As shown in Table 7, the model was statistically significant, F (2, 324) = 88.75, *p* < 0.001, and explained 35.4% of the variance in TL (*R* = 0.595, *R*^2^ = 0.354, adjusted *R*^2^ = 0.350).

**Table 7:** Multiple regression predicting technology literacy.

Predictor	*B*	SE	*t*	*p*	95% CI LL	95% CI UL	β
Intercept	0.964	0.179	5.38	< 0.001	0.611	1.316	—
L2 resilience	0.288	0.053	5.45	< 0.001	0.184	0.392	0.283
L2 engagement	0.411	0.054	7.68	< 0.001	0.306	0.516	0.399

*N* = 327. *R* = 0.595, *R*^2^ = 0.354, adjusted *R*^2^ = 0.350, *F*_(2, 324)_ = 88.75, *p* < 0.001. CI, confidence interval.

Both predictors were statistically significant. L2 resilience positively predicted TL [*B* = 0.288, SE = 0.053, *t* = 5.45, *p* < 0.001, 95% CI (0.184, 0.392), β = 0.283]. L2 engagement also positively predicted TL [*B* = 0.411, SE = 0.054, *t* = 7.68, *p* < 0.001, 95% CI (0.306, 0.516), β = 0.399]. The standardized coefficients indicate that L2 engagement was the stronger predictor in the model, although L2 resilience also made a significant independent contribution. However, because the model explained 35.4% of the variance in TL, the findings should be interpreted as evidence of meaningful but partial prediction rather than a comprehensive explanation of CSL learners' TL.

### Robustness checks

Robustness checks supported the robustness of the main findings. Using HC3 robust standard errors, both L2 resilience (*B* = 0.288, robust SE = 0.059, *p* < 0.001) and L2 engagement (*B* = 0.411, robust SE = 0.058, *p* < 0.001) remained significant predictors of TL. Bootstrap percentile confidence intervals based on 5,000 resamples also excluded zero for both predictors: L2 resilience, 95% CI [0.173,0.400]; L2 engagement, 95% CI [0.304,0.523] (see Table 8).

**Table 8:** Bootstrap confidence intervals for regression coefficients.

Predictor	*B*	Boot SE	Boot 95% CI LL	Boot 95% CI UL	β	β 95% CI LL	β 95% CI UL
Intercept	0.964	0.175	0.622	1.315	—	—	—
L2 resilience	0.288	0.059	0.173	0.400	0.283	0.171	0.392
L2 engagement	0.411	0.056	0.304	0.523	0.399	0.297	0.502

A sensitivity analysis excluding the four exact duplicate cases produced the same substantive pattern. The model remained statistically significant, *F*_(2, 320)_ = 75.33, *p* < 0.001, explaining 32.0% of the variance in TL (*R*^2^ = 0.320, adjusted *R*^2^ = 0.316). L2 resilience (*B* = 0.276) and L2 engagement (*B* = 0.397) both remained positive predictors, with L2 engagement continuing to show the stronger association with TL.

In summary, the quantitative findings indicate that CSL learners in this sample reported a moderate level of TL and that both L2 resilience and L2 engagement were significant positive predictors of TL. Among the two predictors, L2 engagement showed the stronger predictive effect. At the same time, the model explained a meaningful but partial proportion of variance in TL, suggesting that other learner- and context-related factors may also contribute to CSL learners' technology-mediated learning capacity. These quantitative findings provide the basis for the following qualitative analysis, which further examines how learners use technological tools to navigate difficulties and sustain out-of-class Chinese learning practices.

## Results of interview

### Theme 1: differentiated uses of technology in out-of-class Chinese Learning

All six interviewees described using technology to support their autonomous Chinese learning beyond the classroom. Their accounts suggested that technology use was not limited to accessing digital resources, but also involved selecting tools according to learning needs, Chinese proficiency, and the specific problems they encountered during out-of-class learning.

A recurring pattern was that learners with lower or intermediate Chinese proficiency tended to use technology mainly as learning support, especially for listening practice, vocabulary learning, pronunciation, and comprehension. In contrast, higher-proficiency learners described using technology more for evaluating language output, accessing authentic discourse, expanding sociocultural understanding, and supporting academic or professional communication in Chinese.

S1, a second-year university student from Russia, had participated in a one-semester Chinese language exchange program. Although her reading and writing proficiency were relatively strong, her speaking and listening skills, particularly in authentic communicative settings, remained less fluent. She described using Chinese media to create an immersive listening environment outside the classroom:

“First, I dedicate one hour every evening to watching Chinese TV shows and dramas. Initially, I would turn on the Chinese subtitles, but gradually, I tried turning off the subtitles and just listening to the audio. This method not only helped me improve my listening comprehension but also taught me many spoken expressions and new vocabularies.”

By contrast, S4, a French PhD student in sociology at a Chinese university, described using technology to access authentic Chinese-language discussions related to her research interests. For her, technology was not only a tool for language practice, but also a way to observe how Chinese speakers discuss social and academic topics in everyday digital spaces:

“For example, I use Weibo, Zhihu, or Xiaohongshu to see how different groups discuss the topic of ‘foreigners learning Chinese,' and I also participate in some discussions on Douban. For me, this is also a form of Chinese learning, but it's a more socialized and authentic language environment.”

Together, these accounts show that the interviewees used technology in differentiated ways: some used it primarily to compensate for linguistic limitations and increase exposure to Chinese, whereas others used it to participate in more authentic, socially situated, and academically relevant Chinese-language environments.

### Theme 2: goals, fatigue, and persistence in out-of-class Chinese learning

The interviews also showed that learners' use of technology was closely connected to how they managed fatigue, workload, and long-term Chinese learning goals. Several interviewees described out-of-class Chinese learning as difficult to sustain, especially when Chinese study competed with disciplinary coursework, research responsibilities, or daily work. In these accounts, technology functioned as a practical support that helped learners maintain regular exposure to Chinese, organize review, and continue learning even when their motivation fluctuated.

The interviewees' goals appeared to differ according to their learning stage and life context. For learners at lower or intermediate proficiency levels, technology-mediated Chinese learning was often linked to examination preparation, vocabulary memorization, grammar review, and listening practice. For more advanced learners, technology use was more closely connected to maintaining Chinese proficiency, expanding authentic language exposure, and participating in professional or social life in Chinese.

S2, a third-year civil engineering student from Saudi Arabia, described Chinese learning as necessary but difficult to balance with his major coursework. Although he had been studying Chinese for several years, his academic interests were more closely related to engineering modeling and data analysis. At the same time, his coursework, examinations, and scholarship requirements meant that Chinese remained an important academic obligation. He therefore used apps and online courses to support examination-oriented Chinese study:

“Because my primary goal is to pass the HSK Level 4 and Level 5 exams, I mainly use apps and online courses for my review. For instance, I often use a mobile app to memorize vocabulary, and I also look for instructional videos on Bilibili, especially those teaching grammar and listening skills. I usually spend about 40 minutes a day studying Chinese, but the time is not fixed; it's typically after I finish my engineering coursework in the evening.”

In contrast, S6, who had worked in China for 5 years after graduation, described his out-of-class Chinese learning as less examination-oriented and more integrated into everyday life. For him, technology was used not only to maintain workplace communication, but also to access more natural, socially situated, and culturally specific Chinese expressions:

“My out-of-class learning now is actually no longer ‘planned' like it was before; it's more like ‘maintenance and refinement.' I use Chinese every day at work—writing emails, chatting with colleagues—but I know that ‘workplace language' can easily become a habit, so I intentionally look for ‘breakthroughs.' For example, I subscribe to the WeChat official accounts like Renwu and GQ Lab, and I listen to podcasts like Bafen and Gushi FM on Xiaoyuzhou. I like this content because the language is natural, rhythmic, and often includes some slang or dialects.”

Together, these accounts suggest that technology helped the interviewees sustain Chinese learning in different ways: for some, it supported structured review under academic pressure; for others, it provided continued exposure to authentic Chinese use in work, social, and cultural contexts.

### Theme 3: engagement through meaningful, authentic, and manageable technology-mediated practice

The interviews further showed that engagement in out-of-class Chinese learning was shaped by how learners connected technology use with personally meaningful learning activities. Participants described technology-mediated learning not only as a way to complete language tasks, but also as a way to maintain interest, access authentic Chinese contexts, and manage learning fatigue.

S3, a master's student from Japan studying economics at a university in Shanghai, described using financial videos as a way to connect Chinese learning with his academic field. For him, this connection made out-of-class Chinese learning feel more relevant and sustainable:

“I think the most helpful approach for me is watching financial videos and taking brief notes. On one hand, the video content is relevant to my major, which keeps me interested and helps me stick with it; on the other hand, I can learn Chinese vocabulary for expressing economic issues in an authentic context, such as ‘inflation,' ‘consumer confidence,' and ‘structural adjustment.' This learning method, which is related to my academics, makes me feel that studying Chinese is not an extra task but a part of my research.”

S5, a business exchange student from Mexico, emphasized the role of out-of-class experiences in helping him understand pragmatic and cultural aspects of Chinese communication. His account suggested that technology-mediated and real-life learning experiences helped him notice how Chinese is used differently across social and business situations:

“One more thing: I found that out-of-class experiences make me more sensitive to the ‘cultural flavor' of Chinese. For instance, I can now feel the difference between ‘polite talk' and ‘sincere talk,' and I understand what is appropriate to say in different situations.”

Cognitive engagement was also reflected in learners' attention to expression, language style, and culturally appropriate communication. S4 described how long-term exposure to online Chinese comments, podcasts, and authentic written materials helped her improve her academic Chinese writing and develop a more natural sense of expression:

“Furthermore, out-of-class study helped me build a more natural language sense and expressive habits. When I first came to China, my writing for academic papers was always too much like a ‘translation style,' and the sentences were very divisive. But after reading Chinese comments and listening to podcasts for a long time, I started learning to use simpler sentence structures. Now, when I write Chinese academic papers, although it's not as fluent as my mother tongue, I can express complex ideas much more naturally.”

Some interviewees also described fragmented-time learning as a strategy for sustaining engagement. Rather than treating Chinese learning as a single intensive task, they divided learning into smaller and more manageable activities, such as watching short videos, listening to music, reviewing vocabulary, or engaging with relaxing Chinese content after academic work. S1 explained:

“I learned to study in phases: during the day, I focus on academic content, but in the evening, I only study relaxing Chinese, like listening to music or watching short videos. This way, it is both relaxing and provides continuous input.”

Together, these accounts indicate that the interviewees sustained engagement by connecting technology-mediated Chinese learning with academic interests, authentic communication, cultural understanding, and manageable learning routines. These findings help explain why engagement was positively associated with TL in the quantitative results: participants who remained engaged appeared more likely to use technological tools repeatedly, flexibly, and purposefully in their out-of-class Chinese learning.

## Discussion

This study examined CSL learners' technology literacy (TL) in autonomous out-of-class Chinese learning and explored how L2 resilience and L2 engagement predicted and contextualized learners' TL. Overall, the findings suggest that TL should not be understood merely as the ability to operate digital tools. Rather, in language learning beyond the classroom, TL functions as a technology-mediated self-regulated learning capacity, involving resource selection, information evaluation, tool use, problem solving, and sustained participation in digital learning environments. This interpretation is consistent with broader understandings of digital competence, which emphasize problem solving, communication, and purposeful participation in digital contexts (Vuorikari et al., 2022), and with recent work showing the increasing importance of technology-mediated language learning beyond formal instruction (Butler, 2022; Lai et al., 2024).

The quantitative results showed that both L2 resilience and L2 engagement significantly predicted TL, with L2 engagement showing the stronger predictive effect. This finding supports the argument that TL develops through repeated and purposeful learning activity rather than through access to technology alone. Engagement provides the behavioral, emotional, and cognitive basis through which learners continue to use digital tools, interact with authentic materials, and refine their learning strategies. This aligns with previous research that conceptualizes engagement as a multidimensional construct involving behavioral participation, emotional investment, and cognitive effort (Fredricks et al., 2004; Jiang and Peng, 2023). The qualitative findings further explained this relationship: learners who connected technology use with academic interests, authentic communication, cultural understanding, and manageable learning routines appeared more able to sustain technology-mediated Chinese learning beyond the classroom.

L2 resilience also made a significant independent contribution to TL. This suggests that learners' ability to persist through difficulty is relevant to their capacity to use technology effectively in autonomous CSL learning. Out-of-class learning often lacks immediate teacher support, and learners must cope with technical challenges, information overload, language difficulty, and academic fatigue. Prior studies have shown that resilience is closely related to L2 learning achievement and engagement (Chen X. et al., 2025; Liu et al., 2022; Wang et al., 2025). The interview data in this study add contextual detail by showing that learners used technological tools to maintain exposure to Chinese, organize examination preparation, and continue learning despite competing academic or professional demands.

Figure 1 illustrates an interpretive model of the relationship among TL, L2 resilience, and L2 engagement in out-of-class CSL learning. In this model, TL represents the broader technology-mediated learning capacity, while resilience and engagement operate as affective and behavioral supports that help learners transform digital access into sustained learning practice.

**Figure 1:**
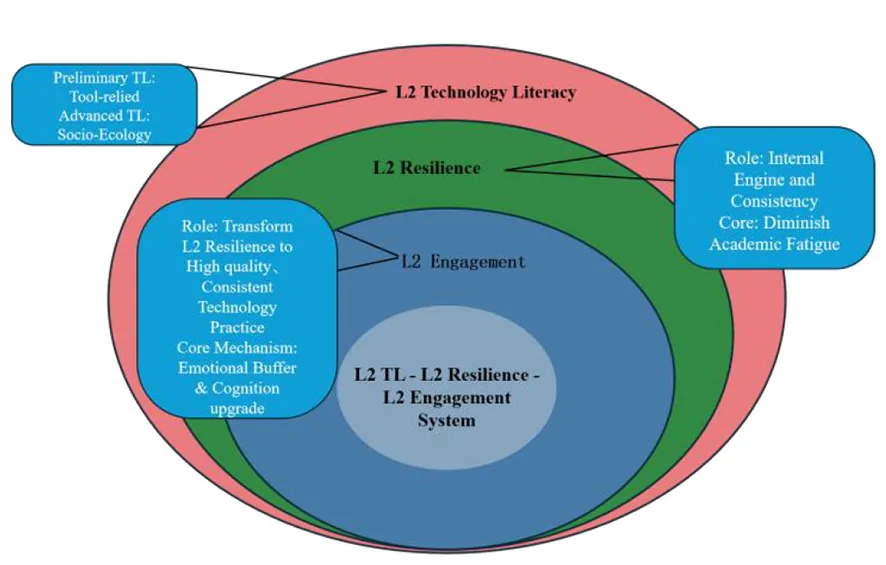
L2 TL—L2 resilience—L2 engagement system.

At the same time, the regression model explained 35.4% of the variance in TL, indicating meaningful but partial prediction. This means that other learner- and context-related factors, such as Chinese proficiency, length of residence in China, prior experience with digital Chinese learning, institutional support, and access to authentic digital communities, may also shape CSL learners' TL. Therefore, the findings should be interpreted cautiously. Rather than claiming that resilience and engagement fully explain TL, this study suggests that they are two important learner-side factors that help explain why some CSL learners are better able to use technology purposefully and sustainably beyond the classroom.

## Conclusion

This study investigated CSL learners' technology literacy (TL) in autonomous out-of-class Chinese learning and examined the predictive roles of L2 resilience and L2 engagement. The findings showed that participants reported a moderate level of TL, suggesting that CSL learners in this sample had some ability to use digital resources for Chinese learning, although this capacity was not uniformly strong. The regression results further indicated that both L2 resilience and L2 engagement significantly and positively predicted TL, with L2 engagement showing the stronger predictive effect. The qualitative findings provided additional explanation by showing how learners used technological tools to support examination preparation, maintain exposure to Chinese, access authentic digital communities, and connect Chinese learning with academic, professional, and everyday life needs.

These findings suggest two pedagogical implications. First, CSL instructors should provide more explicit guidance on how learners can select and combine technological tools for out-of-class Chinese learning. Rather than assuming that learners can naturally benefit from digital resources, teachers can model how different tools support different learning purposes, such as vocabulary review, listening practice, pronunciation support, academic writing, sociocultural understanding, and authentic communication. Such guidance may be particularly useful for lower- and intermediate-proficiency learners, who often rely on technology to compensate for limited linguistic resources.

Second, instructors and institutions should help learners transform technology-mediated Chinese learning into sustained and meaningful practice. For advanced learners, support should move beyond tool operation and focus more on authentic participation in Chinese-language digital spaces, such as academic forums, social media platforms, podcasts, professional communities, and culturally situated online discussions. In this way, TL can become part of learners' broader self-regulated learning capacity, helping them connect Chinese learning with personal goals, disciplinary interests, and social participation.

This study also has several limitations. First, although the quantitative sample was suitable for regression analysis, the study did not conduct stratified analyses by variables such as Chinese proficiency, length of residence in China, academic major, institutional context, or prior experience with digital Chinese learning. Second, the qualitative phase included only six interviewees, so the interview findings should be understood as explanatory and illustrative rather than statistically representative. Third, the cross-sectional design limits the extent to which developmental or causal claims can be made. Future research should include more fine-grained learner background variables, recruit a larger and more diverse qualitative sample, and adopt longitudinal designs to examine how CSL learners' TL changes over time.

## Data Availability

The datasets presented in this article are not readily available because public sharing of the participant-level questionnaire and interview data was not covered by the informed consent and ethics approval. Requests to access de-identified data should be directed to the corresponding authors and will be considered in accordance with applicable ethical and institutional requirements.
