# Supplementary material for: Technology-mediated Chinese learning beyond the classroom: the role of L2 resilience and L2 engagement
Source: Front Psychol. 2026 Sep 23;17:1920084. doi: 10.3389/fpsyg.2026.1920084 (PMC13642584; doi:10.3389/fpsyg.2026.1920084)
Supplement: Supplementary file 1 [file Supplementary_file_1.docx]

**Supplementary Material**

Supplementary Material Caption: Questionnaire items and semi-structured interview questions used in the study. The supplementary file contains no author list, affiliations, or correspondence details.

Note: Items Q6-Q8 in the Technology Literacy scale were reverse-coded in the main analysis using 6 - x. Higher scores indicate higher levels of the measured construct after reverse coding where applicable.

**Appendix A. Questionnaire**

**A1. Technology Literacy**

1. I am proficient in using search engines (such as Baidu, Google) and office software (such as Word, PPT) to search and complete tasks related to Chinese learning.

2. I can creatively use online resources to assist in my Chinese learning. For example, I can search for multimedia materials such as pictures and videos to create Chinese learning reports or presentations.

3. I can use the information technology knowledge I have mastered to solve practical problems encountered in Chinese learning.

4. I can clearly express my views or ideas on Chinese learning on the Internet through various media forms such as text, audio, and pictures.

5. When using technology to learn Chinese, it was difficult for me to control the amount of time I spent online. [Reverse-coded]

6. When I use the internet to learn Chinese, I can easily be distracted by other irrelevant information (such as social media and news). [Reverse-coded]

7. When I study Chinese outside of class, I usually have no plan for which technology tools to use or what content to learn. [Reverse-coded]

8. When browsing Chinese online information, I can keep a clear head and identify unreliable or harmful information.

9. I clearly understand that Chinese learning resources on the Internet contain both opportunities and risks (such as outdated information and inconsistent quality).

10. If I have a disagreement with others about Chinese learning on the Internet, I will communicate rationally instead of making emotional accusations.

11. I believe we should respect different online cultures around the world and reflect this respect in Chinese online communication.

12. I am immune to bad Chinese information on the Internet (such as false advertising and misleading content) and will not be affected by it.

13. I am good at using the Internet to expand my Chinese learning social circle (such as meeting language partners, joining learning communities, and using chatbots).

14. I was able to make new friends and communicate in Chinese through the Internet (such as language exchange apps and learning forums).

15. I am good at using social media (such as WeChat, Weibo, and Bilibili) to showcase my Chinese learning achievements or personal style.

16. I can find the Chinese learning methods and resources that best suit me through the Internet.

17. I can obtain information through the Internet and organize and filter out useful content to complete Chinese learning tasks (such as writing essays and making reports).

18. I can effectively use the Internet to improve myself, especially in learning and improving my Chinese language skills.

**A2. L2 Resilience**

1. Despite the pressures of learning Chinese, I think these pressures have made me a more motivated person.

2. Despite the difficulties, I think I can achieve the desired Chinese learning goals I have in mind through hard work.

3. I can still stay focused in the face of the pressure to learn Chinese.

4. Although there were times when I did not achieve my desired Chinese grades, I was not discouraged.

5. By learning Chinese outside the classroom, I reached my Chinese learning goals.

6. I can adapt to changes in learning Chinese.

7. By learning Chinese outside the classroom, I think my life has become more fulfilling.

8. By learning Chinese outside the classroom, I have become more confident in the matter of learning Chinese.

9. Learning Chinese outside of class makes me feel happy.

10. Learning Chinese outside of class has become a source of joy in my life.

**A3. L2 Engagement**

1. Outside of class, I can effectively use various apps or online tools (such as dictionaries, flashcards, and corpora) to learn Chinese.

2. I will make plans for my Chinese learning outside of class (such as daily vocabulary, weekly reading tasks).

3. When I study Chinese outside of class, I am able to concentrate and try not to be distracted by things like social media.

4. I learn about Chinese through entertainment, such as watching Chinese movies and TV shows and listening to Chinese songs.

5. When I encounter Chinese content that I do not understand, I will actively use online resources to find the answers.

6. I think it is valuable to spend time learning Chinese outside of class.

7. I have a strong interest and curiosity in Chinese language and culture.

8. I feel happy when I learn a new Chinese expression or understand a grammar point.

9. Even if there is no mandatory requirement, I am willing to take the initiative to spend time to learn Chinese.

10. I find great enjoyment in learning Chinese using technological tools.

11. In my extracurricular Chinese studies, I was able to achieve the learning goals I set for myself.

12. I feel a sense of accomplishment when I complete a challenging task in Chinese (such as watching a video without subtitles or writing a diary entry).

13. I believe that through continued extracurricular learning, my Chinese level will continue to improve.

14. I will strive to perform at my best in online language exchanges or writing exercises.

15. I will take the initiative to post or communicate with people in Chinese on online language communities (such as Douyin and Xiaohongshu).

16. I am willing to actively participate and express my opinions during online group learning or discussions.

17. When others communicate with me online in Chinese, I respond positively.

18. I set aside time almost every day to study Chinese outside of class.

19. I will attend online Chinese tutoring classes or language exchange activities on time.

20. I will complete the extracurricular Chinese learning tasks set by myself or assigned by teachers on time.

**Appendix B. Semi-Structured Interview Questions**

1. What technological tools do you usually use to learn Chinese outside of class?

2. Do you feel a lot of pressure when learning Chinese outside of class?

3. Does extracurricular Chinese learning provide positive feedback to your in-class Chinese learning?

4. What difficulties have you experienced when learning Chinese outside of class?

5. Do you think learning Chinese outside of class is a worthwhile investment?

6. What roles do you think society, schools, and teachers should play in your future independent use of technology to learn Chinese?
